# Overview of external reference pricing systems in Europe

**DOI:** 10.3402/jmahp.v3.27675

**Published:** 2015-09-10

**Authors:** Cécile Rémuzat, Duccio Urbinati, Olfa Mzoughi, Emna El Hammi, Wael Belgaied, Mondher Toumi

**Affiliations:** 1 Creativ-Ceutical, Paris, France; 2 Creativ-Ceutical, Milan, Italy; 3 Faculty of Medicine, Public Health Department, Research Unit EA 3279, University Aix-Marseille, Marseille, France

**Keywords:** external reference pricing, European Union, pharmaceutical expenditure, health policy, cost containment

## Abstract

**Background and objectives:**

External reference pricing (ERP) is a price regulation tool widely used by policy makers in the European Union (EU) Member States (MS) to contain drug cost, although in theory, it may contribute to modulate prices up and down. The objective of this article was to summarise and discuss the main findings of part of a large project conducted for the European Commission (‘External reference pricing of medicinal products: simulation-based considerations for cross-country coordination’; see www.ec.europa.eu/health/healthcare/docs/erp_reimbursement_medicinal_products_en.pdf) that aimed to provide an overview of ERP systems, both on processes and potential issues in 31 European countries (28 EU MS, Iceland, Norway, and Switzerland).

**Methods:**

A systematic structured literature review was conducted to identify and characterise the use of ERP in the selected countries, to describe its impact on the prices of pharmaceuticals, and to discuss the possible cross-country coordination issues in EU MS. This research was complemented with a consultation of competent authorities’ and international organisations’ representatives to address the main issues or uncertainties identified through the literature review.

**Results:**

All selected countries applied ERP, except the United Kingdom and Sweden. Twenty-three countries used ERP as the main systematic criterion for pricing. In the majority of European countries, ERP was based on legislated pricing rules with different levels of accuracy. ERP was applied either for all marketed drugs or for specific categories of medicines; it was mainly used for publicly reimbursed medicines. The number of reference countries included in the basket varied from 1 to 31. There was a great variation in the calculation methods used to compute the price; 15 countries used the average price, 7 countries used the lowest price, and 7 countries used other calculation methods. Reported limitations of ERP application included the lack of reliable sources of price information, price heterogeneity, exchange rate volatility, and hidden discounts. Spill-over effect and downward price convergence have often been mentioned as ERP's consequences leading to pricing strategies from pharmaceutical companies.

**Conclusion:**

While ERP is widely used in Europe, processes and availability of price information vary from one country to another, thus limiting ERP implementation. Furthermore, ERP spill-over effect is a major concern of pharmaceutical firms leading to implementation of the so-called ‘launch sequence strategies’.

Since the 1990s, a large number of cost-containment measures have been adopted by the European Union (EU) Member States (MS) to overcome the ever-growing pharmaceutical expenditure, in particular the costs borne by public payers (1). Despite these measures, public pharmaceutical expenditure in the out-patient sector has increased by 76% between 2000 and 2009 in EU countries (approximately from €260 to €340 in purchasing power standard per capita) (1).

With the global financial crisis of 2008, an increased pressure was exerted on most states to lower their budget, and health expenditures became a major target of cost-containment efforts: from 2010 to 2011, 89 measures were implemented in 23 countries to contain pharmaceuticals public expenditure (1). The largest number of these measures were implemented in Iceland, the Baltic States (Estonia, Latvia, and Lithuania), Greece, Spain, and Portugal (1), and consisted mainly in price reductions and changes in co-payments, medicines’ value added tax (VAT) rates as well as distribution margins. Among price regulation measures, external reference pricing (ERP; or international reference pricing, IRP) has also been widely used by policy makers to contain drug costs, although in theory, it may contribute to modulate prices up and down (1, 2). ERP was defined by the World Health Organization (WHO) Collaborating Centre for Pricing and Reimbursement Policies as *The practice of using the price(s) of a medicine in one or several countries in order to derive a benchmark or reference price for the purposes of setting or negotiating the price of the product in a given country* (3). As such, changes in a given drug price in one country will influence its price in other countries. Worldwide, non-EU countries (e.g., Brazil, Jordan, South Africa, Japan, Turkey, Canada, and Australia) also apply ERP and often use EU MS as reference countries (4, 5).

Processes of ERP implementation and availability of price information vary from one country to another. Various stakeholders have also expressed several concerns and limitations related to ERP application.

To gain a better understanding of ERP application and potential impacts in Europe, a research providing an overview of ERP systems, both on processes and potential issues in European countries, was conducted. This project was initially performed for the European Commission (6); the present article summarises and discusses the main findings of this research.

## Methods

A systematic structured literature review was conducted to identify and characterise the use of ERP, to describe its impacts on the prices of pharmaceuticals, and to discuss the possible cross-country coordination issues in European countries. The research was complemented with a consultation of competent authorities’ and international organisations’ representatives to address the main issues or uncertainties identified through the literature review.

The research covered all of the 28 EU MS: Austria, Belgium, Bulgaria, Croatia, Cyprus, Czech Republic, Denmark, Estonia, Finland, France, Germany, Greece, Hungary, Ireland, Italy, Latvia, Lithuania, Luxembourg, Malta, the Netherlands, Poland, Portugal, Romania, Slovakia, Slovenia, Spain, Sweden, and the United Kingdom (UK), but also Switzerland, Norway, and Iceland.

### Systematic structured literature review

#### Search strategy

Using the OVID website,^1^ we searched Medline^®^ and Medline^®^ in process, EMBASE^®^, and EconLit for full paper manuscripts published between January 1997 and August 2013.

The search strategy consisted of the following free search terms: Reimburs** decision$.mp. OR reference pric*.mp. OR (Cross countr* adj25 (health* or pric*)).mp. OR (Pric* adj5 benchmark*).mp. OR (Pric* adj10 polic*).mp. OR (Differen* adj5 pric*).mp. OR Pric* set*.mp. OR Pric* regula*.mp. OR maximum allowable cost*.mp. OR (cost control adj5 (pharma* or drug* or medicine*)).mp. OR reference drug* pric*.mp.

To cover most of the publications on ERP, we included publications in English and original publications in the following languages: Italian, French, Spanish, German, Dutch, Danish, Swedish, and Polish.

As ERP can apply to all pharmaceutical products on the market, all medicinal products were included in the review. Publications dealing with devices, vaccines, and diagnostics were excluded, as well as papers dealing with internal reference pricing or referring to countries which are not listed among the selected countries.

A search of abstracts presented in the past 3 years at the International Society for Pharmacoeconomics and Outcome Research (ISPOR) and the Health Technology Assessment International (HTAi) conferences was also carried out, respectively, via the EMBASE^®^ database and the official HTAi website.^2^ The search terms used were ‘reference price’ OR ‘reference pricing’.

Additionally, a search was performed in Google Scholar and official website of organisations such as the WHO,^3^ the Organisation for Economic Co-operation and Development (OECD),^4^ and the European Commission.^5^ The search terms used for the online searches were ‘External reference pricing’, ‘External price referencing’, ‘International price referencing’, ‘International reference pricing’, ‘International price comparison’, ‘International price benchmark’, ‘External price benchmark’, ‘External price linkage’, and ‘International price linkage’.

The literature review was completed with a search on National Health Authorities and parliament websites, as well as a search in press and media and in our internal proprietary database, when relevant.

#### Data collection and extraction

All the references obtained from the searches were imported into a Reference Manager database and duplicate articles were removed from the database.

Articles were screened by two independent reviewers to select relevant articles, according to the defined inclusion and exclusion criteria.

Data were structured in a standard document to capture the main elements characterising ERP systems and to describe ERP application in each country:

- Existence of ERP
- Place of ERP in price setting (main/supportive criterion)
- ERP legal framework
- Composition of the country basket (i.e., countries used as reference)
- Products regulated by ERP
- Reference price calculation methods
- ERP submission process

In a second step, the different elements characterising ERP systems were structured in a questionnaire to allow data analysis and validation of the information via the stakeholder consultation.

#### Data analysis

Based on the extraction and stakeholders’ validation, descriptive statistics and qualitative analysis were performed.

### Stakeholder consultation

The literature review was complemented with a stakeholder consultation conducted between June and August 2013. Written surveys were addressed to:

- Competent authorities’ representatives of the selected countries (i.e., regulatory authorities responsible for human medicines), focusing on specific questions related to ERP processes, and
- Fourteen international organisations’ representatives (industry, patient, doctor, insurance, hospital, wholesaler, and pharmacist representatives), focusing on specific questions related to organisations’ perception of ERP system.

## Results

### Methodology results

#### Systematic search

The search in EMBASE^®^ retrieved 2,441 records, the search in Medline^®^ and Medline^®^ in process retrieved 2,294 records, and the search in EconLit retrieved 407 records, for a total of 5,142 records. A total of 1,741 duplicates were removed, leaving 3,401 titles and abstracts that were reviewed.

The search in conference abstracts retrieved 45 ISPOR abstracts and 4 HTAi abstracts. There were no duplicates, so a total of 49 conference abstracts were reviewed.

The search in Google Scholar retrieved a total of 627 records. There were 88 duplicates, leaving a total of 539 abstracts to be reviewed.

**Fig. 1:**
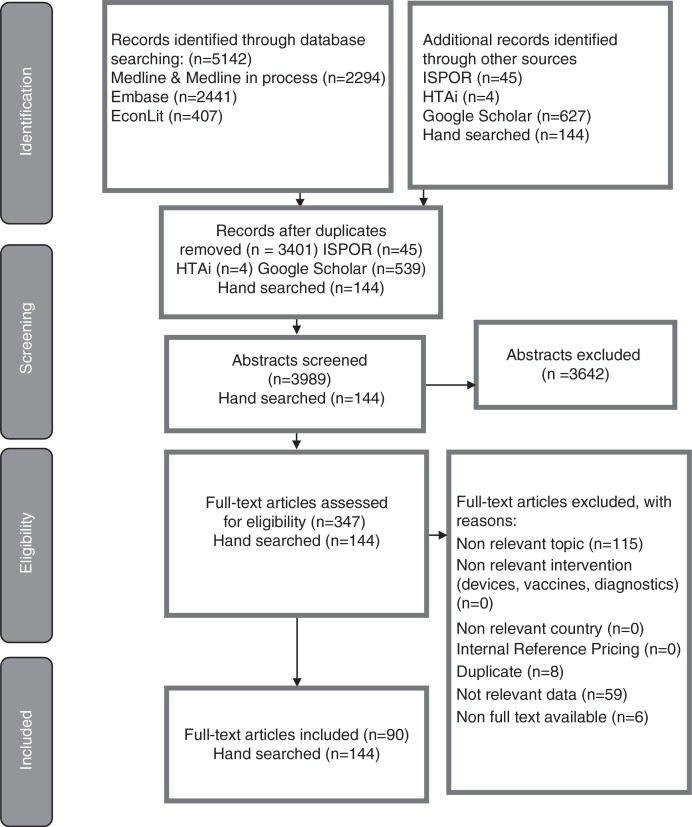
PRISMA diagram of literature search.

Out of the total 3,989 titles and abstracts that were reviewed, 347 were included and 3642 were excluded, and of the 347 papers ordered for full paper review, 6 papers were not available. Reasons for inclusion and exclusion are detailed in Fig. 1. In the end, 90 papers were included for the data extraction and 144 references of interest were retrieved via hand searches in other databases (Fig. 1).

#### Stakeholder consultation

In total, the survey was completed by 20 competent authorities’ representatives from Austria, Belgium, Cyprus, Czech Republic, Finland, Hungary, Iceland, Italy, Latvia, Lithuania, Malta, Norway, Poland, Portugal, Slovakia, Slovenia, Spain, Sweden, Switzerland, and the UK.

Nine competent authorities’ representatives did not reply (Bulgaria, Estonia, France, Germany, Greece, Ireland, Luxembourg, the Netherlands, and Romania).

Croatia was not in the position to reply due to local difficulties in legislation interpretation.

Denmark did not accept the invitation to participate in the study.

As for international organisations’ representatives, six responded to the survey: the European Federation of Pharmaceutical Industries & Associations (EFPIA), the European Generic medicines Association (EGA), the European Self-Medication Industry (AESGP), the European Patients Forum (EPF), the Pharmaceutical Group of the European Union (PGEU), and the European Hospital and Healthcare Federation (HOPE).

Three international organisations stated that they were not directly involved with ERP regulations.

Five international organisations did not reply (Table 1).

**Table 1:** Representatives involved in the stakeholder consultation

Competent authorities’ representatives	International organisations’ representatives
Austria Belgium Cyprus Czech Republic Finland Hungary Iceland Italy Latvia Lithuania Malta Norway Poland Portugal Slovakia Slovenia Spain Sweden Switzerland UK	European Federation of Pharmaceutical Industries & Associations (EFPIA) European Generic medicines Association (EGA) European Self-Medication Industry (AESGP) European Patients Forum (EPF) Pharmaceutical Group of the European Union (PGEU) European Hospital and Healthcare Federation (HOPE)

### ERP processes in Europe

#### Application and use

**Fig. 2:**
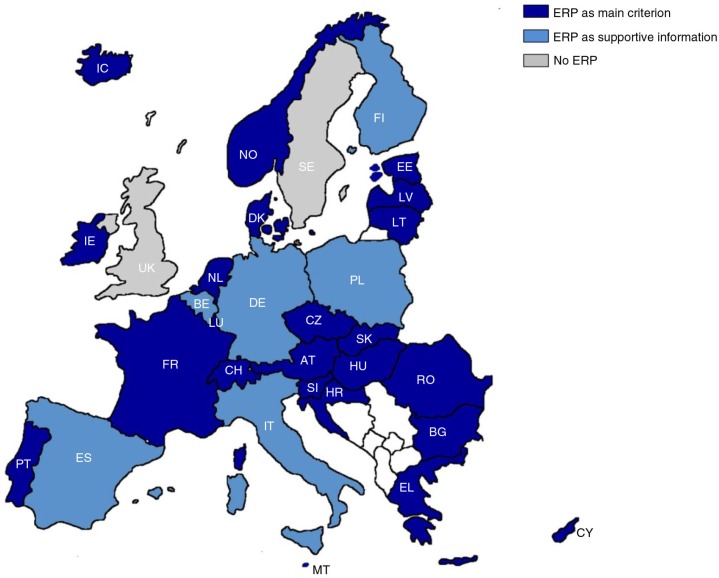
Overview of ERP across Europe (2013).

ERP is widely applied in Europe. As of August 2013, all selected countries, except Sweden and the UK, used ERP (Fig. 2). Denmark and Sweden stopped ERP, switching to internal reference pricing and value-based pricing, respectively. However, Denmark reintroduced ERP in 2009, only for new medicines in the hospital sector, and Sweden was expected to switch back to ERP in 2014, but, to date, this has not been implemented yet (7, 8).

In the majority of the countries (23 out of 31), ERP was used as main systematic criterion when setting the price of a new drug. Only Belgium, Finland, Italy, Poland, Spain and Germany, used ERP as supportive information (Fig. 2).

- In Belgium, ERP was used as supportive of the pricing decision, but also as a criterion for price cuts, which were introduced in the 2013 healthcare budget for reimbursed patented medicines that have been at least 5 years on the market. For these drugs, prices were compared to those in six European countries (Austria, Finland, France, Germany, Ireland, and the Netherlands).
- In Finland, ERP was used as one criterion among many others when approving the ‘reasonable wholesale price’.
- In Germany, Italy, and Poland, ERP was used as additional information during pricing negotiation of reimbursed medicines. It can be noted that, in the past, Italy was using ERP as the main criterion for the pricing of reimbursed medicines.
- In Spain, ERP was used to control the price of medicines for which there are no alternatives available on the Spanish market.

### National legal framework

In the majority of European countries using ERP for setting the price of pharmaceuticals, ERP was based on legislated pricing rules. In Spain, even if ERP was no longer mentioned in the law since the Decree law 16/2012, ERP still conformed to criteria of the Inter-ministerial Pricing Committee. ERP was sometimes part of agreements, such as in:

- France: Framework agreement between the Healthcare Products Pricing Committee and the pharmaceutical companies (*Accord Cadre entre le Comité Economique des Produits de Santé et les Entreprises du Médicament*)
- Ireland: Framework agreement between the Irish Pharmaceutical Healthcare Association Ltd and the Department of Health and the Health Service Executive
- Denmark: Agreement between the Danish government and the Danish Association of the Pharmaceutical Industry (*Aftale om prisreduktioner og loft over priserne for sygehusforbeholdte lægemidler i perioden 1. January 2013–31. December 2015*)

Overall, depending on the country and the use of ERP (main or supportive criterion), ERP methodologies were described in national pricing legal frameworks with different levels of accuracy. Portugal and Austria are two examples of countries for which ERP procedures were well detailed within their pricing regulations. ERP rules were substantially less detailed for Germany or Estonia (Supplementary Table 1).

### Scope of ERP

Except in Luxembourg, where ERP applied for all marketed drugs, ERP was only used for setting the price of specific categories of medicines, such as publicly reimbursed medicines, prescription-only medicines, or innovative medicines. In most cases, ERP was used for publicly reimbursed medicines (16 countries: Austria, Croatia, Czech Republic, Estonia, Finland, France, Germany, Ireland, Italy, Latvia, Lithuania, Malta, Poland, Slovakia, Slovenia, and Switzerland). In Estonia, France, and Germany, ERP applied only for reimbursed innovative medicines. The application of ERP for in-patent or off-patent medicines was not always specified, but reportedly less countries used ERP for off-patent drugs (Austria, Bulgaria, Croatia, Czech Republic, Iceland, Italy, Latvia, Lithuania, the Netherlands, Poland, Romania, Slovakia, and Slovenia).

In most of the countries, no specifications were made as to the application of ERP to in-patient and/or out-patient pharmaceuticals. This information was only stated for Denmark which applied ERP for hospital-only medicines, Portugal which excluded hospital-only medicines from its ERP system, Austria which applied ERP for out-patient pharmaceuticals, and the Netherlands which applied ERP for all outpatient drugs, as well as for high-cost medicines and orphans drugs for in-patient care (Supplementary Table 1).

### Composition of the country basket

The number of reference countries included in the basket varied greatly from one country to another: from one in Luxembourg to 31 in Hungary and Poland (Table 2). While most countries’ basket included EU countries only, the baskets of Hungary, Denmark, Poland, and Finland included also non-EU countries (Iceland, Norway, Switzerland, and Liechtenstein).

The country of origin^6^ was used as reference in Luxembourg and Estonia. In Belgium, where ERP is used as supportive criterion, the methodology reported to set prices was either the average price in the reference countries (26 EU MS) or the price in the country of origin. In Cyprus, Lithuania, and Romania, the price in the country of origin was also used when it was not available for the reference countries.

The most referenced countries were France (19 countries), followed by the UK and Germany (17 countries), Austria, Spain and Slovakia (16 countries), Belgium, Denmark, Finland, the Netherlands, and Italy (15 countries). The least referenced countries were Croatia, which entered the EU in July 2013 (5 countries), and non-EU countries: Switzerland (2 countries), Iceland (3 countries), and Norway (6 countries).

### Price calculation and selection of reference products

ERP regulations were usually described in countries’ legal frameworks; however, the accuracy of this description differed from one country to another. Furthermore, the rules that applied to the choice of the reference products were not always clearly described (e.g., generics, non-reimbursed drugs, out-patient/hospital-only drug, different pack size, different dosages, and different pharmaceutical forms) (Supplementary Table 1) (9, 10).

#### Main calculation methods

The reference price calculation methods were unclear in some cases and differed across countries. The two main calculation methods were the average price and the lowest price. The average price of reference countries was used in Austria, Belgium, Cyprus, Denmark, Iceland, Ireland, Portugal, Switzerland, and the Netherlands. The average of the three or four lowest prices of all countries in the basket was used in Greece, Norway, Slovakia, and Czech Republic. The lowest price among all reference countries was used in Bulgaria, Hungary, Italy, Romania, Slovenia (for original drugs and biosimilars), and Spain. Luxembourg used the price of the country of origin; pharmaceutical's price could not be greater than the price granted by the competent authority of the country of origin. France, with only four countries in its basket, applied similar prices to those in the reference countries.

In Malta two ERP systems, characterised by different rules, were used: one for the private market and another for public sector medicines. For instance, for the public sector, the price was set based on the average wholesale price of the basket, whereas, for the private sector, an algorithm was used for price calculation.

#### Alternative calculation methods

When there was no price available in one or more of the reference countries or when the prices were not approved in all reference countries, some countries estimated the price based on the reference countries where the price was approved; the price of the drug was then revised when it became available in an additional country. In some countries, the price was set only if a comparable drug was marketed in a minimum number of countries, for example, 2 countries in Croatia and The Netherlands, 3 countries in Czech Republic, Greece and Hungary, and 12 countries in Austria. Other countries, such as Bulgaria, Croatia, and Cyprus, set the price using the same method but alternative reference countries. In Romania, the price from the country of origin shall be considered when no price was set in the 12 countries of the basket.

**Table 2:**
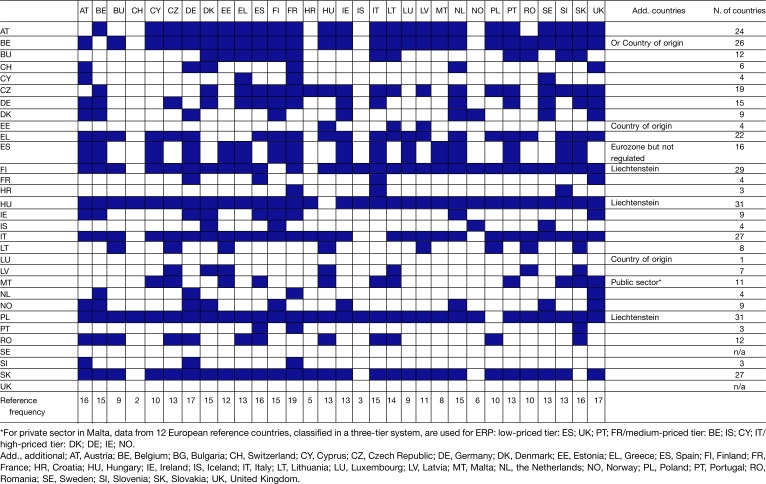
Overview of country baskets in Europe (2013)

#### Price level

In most cases, the ex-factory price was the reference price used to calculate the ‘ERP’ price (17 countries), followed by the pharmacy purchasing price (PPP). The pharmacy retail price (PRP) was used only in two countries: Luxembourg and Malta (public sector). Italy used ex-factory prices, PPP or PRP depending on the information provided by the pharmaceutical company. In Latvia, ERP was applied at ex-factory price and/or PPP level depending on whether the drug was imported or not.

#### Reference product selection

A product that was not reimbursed in a reference country could still be used as reference by some countries (e.g., Austria, Belgium, and Portugal). In general, the branded version was selected for reference purposes even if the generic form was available in a reference country.

When different dosages and pack sizes were approved at different prices in the reference countries, the same or closest pack size or dosage was generally used as reference. In some countries, such as Belgium, Hungary, and Iceland, when the pharmaceutical formulation of a drug in the reference country was different from the formulation approved in the referencing country, the different pharmaceutical formulation was considered only if it was similar to the approved one (e.g., oral solid forms such as capsule versus tablet could be compared to each other but not to injectable forms). Other countries, such as Latvia, Portugal, and Slovakia, did not take into account a different formulation for ERP.

#### Price re-evaluation

Prices could be re-evaluated on a regular basis after the initial price has been set. The frequency and process of reviewing prices differed between countries. Revision frequencies varied from every 3 months (Greece) to every 5 years (Finland and France). In 2012, Ireland performed a downward price realignment based on the currency-adjusted average ex-factory price of the drug in reference countries.^7^ The Norwegian Medicines Agency revaluates annually the maximum price of 250 active ingredients with the highest turnover to ensure that the maximum prices reflect the changes in European prices. In Slovenia, prices were revised twice a year in case changes in the price of reference countries occurred.

### Limitations and potential consequences related to ERP

#### Limitations related to ERP

Even if ERP is a widely accepted and used cost-containment tool, several limitations to its methodology were reported.

First, ERP is characterised by a ‘path dependence’, which means that the observed price levels are influenced by the rules of the systems itself (e.g., country selection, price taken from the basket, and revisions dates) and other aspects of the market, such as health needs, income and healthcare costs, as well as their fluctuations across countries are ignored (11–13).

Furthermore, ERP implementation remains limited due to the lack of available information on drugs’ prices:

- Limited access to prices in EU MS (price unavailability, difficulties in identifying and obtaining relevant data sources) (8).
- Price heterogeneity (e.g., ex-factory prices, PPPs, PRPs) making the price comparison difficult (price derived from calculation, proxy of true price) (5, 12).
- Publicly available prices are often facial prices that do not take into account the managed entry agreements, as these are often confidential (5, 8, 12, 14, 15).
- Lack of transparent price databases that may lead to mistakes in published prices and thus distort ERP-based systems (such as recently seen in Greece where published prices were miscalculated; lower prices than prices obtained if ERP rules had been properly applied).^8^ However, this may be an exceptional case.
- ERP-based price revisions occurring on irregular basis after the initial price has been set, price reductions in reference countries are not automatically translated into price decreases in referencing countries (2).
- Exchange rate volatility affecting prices denominated in local currencies (2, 12). In Switzerland, the reference price is based on Eurozone MS (Austria, France, Germany, and the Netherlands) and non-Eurozone MS (Denmark and the UK). Swiss drug prices have fallen quickly towards the reference basket average over the past 5 years. The appreciation of the Swiss Franc makes foreign prices cheaper and leads to further downward pressure on Swiss ones (16). Furthermore, countries referring to non-Eurozone countries do not always disclose the currency rates used at the time of the calculation, leading to prices miscalculation in other countries.

Additionally, identifying the same medicine in other countries can be challenging due to different commercial names, pharmaceutical formulations, dosages, and pack sizes. This has appeared to be a tactic used by manufacturers to limit opportunities for ERP (2, 5, 15, 17).

Moreover, the various rules adopted by countries to address these specificities raise a concern in terms of representativeness by generating incorrect measures of price differences across countries. For example, as the average pack size can vary significantly across countries, basing the price comparisons on identical pack size would imply to exclude some reference countries, but also to ignore the representativeness of the matching pack size for the price level in the reference countries (18, 19).

### Potential consequences of ERP

#### Spill-over effects and price convergence

The real impact of ERP policy is still not well understood (8, 20). Concerns due to its spill-over effects on other countries have been expressed by the industry (21). ERP is often argued to lead to a downward price convergence. In fact, ERP might lower prices when a MS uses the lowest price in the country basket rather than the average price, or because of currency fluctuations. Due to the wide application of ERP, a low price for a new product in a given market might affect manufacturer's pricing strategies elsewhere and could lead to parallel trade (1, 2, 5, 8, 12, 14).

However, two recent studies on prices of medicines suggested that no substantial reduction in international price differences occurred within EU countries (22, 23). Indeed, a first study looked at over 1,000 prescription drugs in 36 therapeutic categories in 30 countries (EU and non-EU countries) over a 12-year period (1993–2004) to assess whether price dispersion decreased in the EU (where parallel trade is permitted) and non-EU countries (where parallel trade is not permitted). The results showed that about half of the price differentials exceeded 50% in both EU and non-EU countries over time, and price distributions in the EU did not show a dramatic change with the adoption of parallel trade (23). In the second study, prices of 10 on-patent medicines of 15 European countries over 5 years (2007, 2008, 2010, 2011 and 2012) were analysed to assess whether ex-factory prices of on-patent medicines in Western European countries have converged over a recent period of time. A price divergence between 2008 and 2012 was shown (24). This divergence was driven by two countries, Germany (up to 27% more expensive than the average) and Greece (up to 32% cheaper than the average), whereas all other countries had stable prices, centred on the country average. Thus, this study supported a trend for convergence (price close to the country average), with a substantial difference between the lowest price country and the highest price country (24). The authors underlined the need for further research with larger sample size including prescribing data and Eastern European countries (24).

#### Patient access to medicines

ERP has become an incentive for pharmaceutical companies to adopt international pricing strategies. Launch sequence strategies are used to delay or avoid launching new drugs in countries with potential lower prices, especially if they are small markets referenced by countries with larger markets (1, 5, 12, 14, 15). For example, there is evidence that pharmaceutical companies systematically delayed dossier submission in Belgium in order to avoid the Belgian price, usually not in the highest EU range (included in the third group of countries that have price levels between 0 and 15% higher than the EU25 average, from 2005 Eurostat survey) (24, 25).^9^

There is also evidence that the widespread use of ERP determines a circular pricing (the more countries are used as reference countries, the less clear it becomes which countries’ prices are the reference). Price revisions in one country may, at least in theory, trigger a sequence of circular price revisions, further contributing to a strategic launching of a new drug (2).

It is however difficult to assess to what extent strategic launching used to limit ERP spill-over effects is delaying the launch in low-prices countries, as other factors are usually simultaneously present (i.e., parallel trade) (5, 12).

#### Affordability

Although ERP aims to achieve a better control of prices and faster price erosion, it might also induce a vicious effect, such as leading pharmaceutical companies to increase the target price in order to avoid both negative impact on the company's revenues of ERP and parallel trade (1, 2, 5). Carone et al. (15) noted that pharmaceutical prices reported to local purchasing power remain higher in countries with lower absolute price levels of pharmaceuticals (e.g., Poland, Romania, Bulgaria) versus countries with higher absolute price levels (e.g., Germany, Denmark, Ireland, and Italy), thus impacting country's affordability (2).

For example, it was reported during the stakeholder consultation that ERP might lead to product shortage in countries referencing the lowest price, due to discontinuations and parallel export, as illustrated with Bulgaria where about 200 products (strengths, pack sizes, and chemical entities) were withdrawn from the market in 2012.

#### Industry revenue and sustainability

Price convergence reported through ERP-based systems has been argued to discourage incremental innovation from pharmaceuticals companies by reducing revenues and therefore the potential for research and development investment (14, 22, 26). On the contrary, differential pricing (DPR) is described in the literature as a potential effective way for preserving incentives for research and development through higher prices in high income countries. Furthermore, DPR may lead to incremental sales for the pharmaceutical companies, that is, additional revenues (volume) from poorer countries without losing revenues (sales) in richer and less-price-sensitive countries (27, 28).

From the stakeholder consultation, ERP system appears to have a massive negative impact on the pharmaceutical industry competitiveness (off-patent: generic and biosimilar or in-patent medicine industry).

Indeed, from the EGA perspective, and considering the very competitive environment of off-patent medicine market, ERP limits generic medicine industry's potential to enter specific markets by driving down the prices to unsustainable levels. EGA cited the case of the price of the generic medicine olanzapine that dropped by up to 98% in Bulgaria due to application of ERP in Denmark, thus limiting patient access to this medicine in Bulgaria. EGA emphasised that referencing prices in countries where procurement and tendering systems are in place (driving down the prices to ‘unsustainable’ levels) would be detrimental for the generic sector, for patients (availability of affordable generic medicines) and for payers (savings for the national health systems).

From the EFPIA perspective, ERP causes indirect and adverse effects across Europe and beyond, especially in the context of short-term cost-containment measures. The stakeholders illustrated their perspective by providing two studies investigating the impacts of ERP (29, 30). The first study focused on ERP and parallel trade impacts on social welfare and patient access and concluded that ERP and parallel trade created spill-over effects from low price to higher price countries leading to patient access issues in low price markets, with limited benefits to payers and patients in terms of cost-savings for high price markets. These spill-over effects were also likely to have a negative impact on the willingness/potential/capacity to invest in Research and Development, although it was difficult to directly examine this phenomenon. EFPIA illustrated the potential spill-over impact of ERP, in case of price cut, by estimating industry cost following a 10% price drop in Greece in 2011 if all countries re-referencing Greek prices (formal/informal) were included. It was shown that the price drop would have generated losses for the industry of €299 million in Greece, €799 million in Europe, and €2,154 million worldwide (30). The second study looked at the impact of the Swiss drug regulation, focusing on the international impact of price cuts in Switzerland due to ERP (31). The study showed the worldwide spill-over effects from a 10% price reduction in Switzerland if all countries re-referencing Swiss prices (formal/informal) were included. It was shown that the price reduction would reduce industry revenue by €430 million in Switzerland and €495.2 million worldwide (31).

## Discussion and conclusion

While ERP is widely used in Europe to achieve cost-containment, its application and potential impacts remain highly debated. ERP application is limited due to the different price information across countries and varying characteristics of ERP across EU MS, with methodologies evolving over time. ERP spill-over effect within and outside EU MS is a major concern of pharmaceutical firms, leading to implementation of launch sequence strategies and potentially limiting patient access to medicines.

Where ERP is argued to lead to price convergence, DPR is reported as a win–win situation from patients’ and pharmaceutical industry's perspectives by improving access and affordability of medicines while preserving incentives for research and development. Nonetheless, even if ERP is argued to lead to price convergence across Europe, price differences could also result from the different methodologies used for ERP, as well as from other pricing policies in place. These differences could be driven by specific countries only. The price is frequently based on implicit multi-criteria decision, of which ERP is only one among many others. Although pharmaceutical companies try to control ERP, they have little capacity to influence it.

Different ERP policies were proposed using a broad range of attributes, such as to consider as reference countries with comparable GDP per capita, to use the ‘average formula’ instead of ‘lowest formula’, or to take into account the different distribution margins in order to achieve price differentiation across countries. For lower income countries, it has been proposed to agree on a high price list but to negotiate confidential discounts (8, 31, 32).

It might be questioned if the use of ERP, when built on purpose and properly coordinated, may become a tool to achieve DPR and enhance access to innovative expensive medicine in low income markets and therefore the welfare. If different countries use different formula, different price reference, different basket, etc. this might lead to price differentiation. If this is coordinated at EU level, then ERP may become a tool to enhance DPR and welfare of the EU population.

Further research is needed to assess the interactions of both ERP and DPR to maximise cost-containment through ERP, while maximising patient access to medicine through DPR.

## Supplementary Material

Overview of external reference pricing systems in Europe

## Footnotes

1
www.ovid.com/

2
www.htai.org/

3
www.who.int/

4
www.oecd.org/

5
www.ec.europa.eu/

6 It was not clearly defined in the literature if the country of origin was considered the country of the drug manufacturer or the country of the marketing authorisation holder.

7 This was carried out according to the framework agreement between the Irish Pharmaceutical Healthcare Association Ltd and the Department of Health and the Health Service Executive on the Supply Terms, Conditions, and Prices of Medicines.

8 Letter sent by EFPIA Director General to the Head of the European Commission's Task Force for Greece-Extra-territorial impact of erroneous medicine prices published on the website of the Greek Government (2013 Mar 20) (communicated by EFPIA).

9 After the top group consisting of two countries that have significantly higher price levels than the others: Iceland and Switzerland, with price levels being 60% and 87% higher than the EU25 average, respectively, and the second group of most expensive countries (where price levels are between 15% and 30% higher than the EU25 average): Denmark, Germany, Ireland, Italy, and Norway.

## References

[1] Vogler S, Zimmermann N, Leopold C, de Joncheere K (2011). Pharmaceutical policies in European countries in response to the global financial crisis. Southern Med Rev.

[2] European Parliament (2011). Differences in costs of and access to pharmaceutical products in the EU.

[3] The WHO Collaborating Centre for Pricing and Reimbursement Policies, Glossary.

[4] Leopold C, Vogler S, Mantel-Teeuwisse AK, de JK, Leufkens HG, Laing R (2012). Differences in external price referencing in Europe-A descriptive overview. Health Policy.

[5] Espin J, Rovira J, De Labry AO WHO/HAI project on medicine prices and availability-Working paper 1: External reference pricing [updated May 2011].

[6] Toumi M, Rémuzat C, Vataire AL, Urbinati D External reference pricing of medicinal products: Simulation-based consideration for cross-country coordination [updated December 2013].

[7] Bouvy J, Vogler S Pricing and reimbursement policies: Impacts on innovation.

[8] Kaiser U, Méndez SJ, Rønde T, Ullrich H Regulation of pharmaceutical prices: Evidence from a reference price reform in Denmark.

[9] Eurostat Population.

[10] Eurostat GDP.

[11] Kanavos P, Espin J, van der Aardweg S Short- and long-term effects of value-based pricing vs. external price referencing.

[12] Cueni TB International price referencing-is there a “right” way to perform it? Presentation.

[13] Docteur E Value for money and valued innovation: A trade-off or mutually compatible goals? OECD High-Level Symposium on Pharmaceutical Pricing Policy [updated 27 October 2008].

[14] OECD Improving health-system efficiency: Achieving better value for money-Ensuring efficiency in pharmaceutical expenditures: Policies to improve value for money.

[15] Carone G, Schwierz C, Xavier A Cost-containment policies in public pharmaceutical spending in the EU.

[16] The Pharmaletter Swiss pharma market shrank for first time in 2010 [February 2011].

[17] OECD Pharmaceutical pricing policies in a global market [updated September 2008].

[18] Brekke KR, Holmås TH, Straume OR Comparing pharmaceutical prices in Europe. A comparison of prescription drug prices in Norway with nine Western European countries.

[19] Brekke KR, Holmås TH, Straume OR Are pharmaceuticals still inexpensive in Norway? A comparison of prescription drug prices in ten European countries.

[20] Ruggeri K, Nolte E (2013). Pharmaceutical pricing. The use of external reference pricing.

[21] Eucope (European Confederation of Pharmaceutical Entrepreneurs) (2012). Explanatory memorandum. Pharmaceutical prices: Why are there differences between Member States.

[22] Kyle MK, Allsbrook JS, Schulman KA (2008). Does reimportation reduce price differences for prescription drugs? Lessons from the European Union. Health Serv Res.

[23] Leopold C, Mantel-Teeuwisse AK, Vogler S, de Joncheere K, Laing R, Leufkens HG (2013). Is Europe still heading to a common price level for on-patent medicines? An exploratory study among 15 Western European countries. Health Policy.

[24] Eurostat Pharmaceutical products – Comparative price levels in 33 European countries in 2005 –Issue number 45/2007.

[25] Maervoet J, Toumi M Time to market access for innovative drugs in England, Wales, France and Belgium.

[26] Garau M, Towse A, Danzon P Pharmaceutical pricing in Europe: Is differential pricing a win-win solution? Office of health economics.

[27] Yadav P Differential pricing for pharmaceuticals [updated August 2010].

[28] Watal J, Consultant to the WTO Secretariat (2001). Workshop on differential pricing and financing of essential drugs.

[29] Charles River Associates (2012). The implications of international reference pricing and parallel trade on social welfare and patient access.

[30] Charles River Associates (2013). The international impact of Swiss drug regulation. Published by Interpharma, Association of research based pharmaceutical companies in Switzerland (Basel), Novartis International AG (Basel).

[31] EFPIA Improving patient access to innovative medicines. The framework in which differentiated pricing may offer a solution [28 February 2014].

[32] Zoltan K, Annemans L, Garrison LP (2013). Differential pricing of new pharmaceuticals in lower income European countries. Expert Rev Pharmacoecon Outcomes Res.

