# Supplementary material for: Overview of external reference pricing systems in Europe
Source: J Mark Access Health Policy. 2015 Sep 10;3:10.3402/jmahp.v3.27675. doi: 10.3402/jmahp.v3.27675 (PMC4802694; doi:10.3402/jmahp.v3.27675)
Supplement: Overview of external reference pricing systems in Europe [file JMAHP-3-27675-s001.pdf]

Supplementary files

Table S1. Overview of ERP Processes in Europe

Country	Population[i]	GDP per capita, current prices, current purchasing power parities (Euro) 2012[ii]	ERP application	Use of ERP	National legal framework related to ERP	Products regulated by ERP	Number of countries in basket	Price level taken into account for reference purposes	Reference price calculation	Rules adopted in case the price is not approved in all reference countries	Price revisions	Rules to choose pack sizes/dosages/pharmaceutical formulations as reference
Austria	8,443,018 (2012)	36,400	Yes	Main criterion	Yes<br>Law: Austrian Social Insurance Law (ASVG)<br>Decree: Regulation on Procedural Rules for Calculation of the EU average price - 1 October 2005 (according to Art. 351c.6 ASVG)	Reimbursed medicines in out-patient sector (in-patent /off-patent)	24 EU MS	Ex-factory price	Average price of reference countries	Price can be established if drug is marketed in at least half of the EU MS for on-patent pharmaceuticals and in at least two EU MS for generics. Otherwise, a temporary price is determined[1]	No	-When different pack sizes/dosages are approved in the reference countries at different prices, the same pack size/dosage of the assessed drug are used as reference<br>-If the same pack size/dosage are not available, the closest pack size/dosage of the assessed drug are used as reference<br>-If there is no comparable pack size, the average price per unit for all pack sizes marketed in the concerned EU MS is used (Conversion factor: price per unit is considered).<br>-If there is no comparable dosage, different dosage used as reference only when the same reference is not approved in at least 2 countries(Conversion factor: price per unit is considered)<br>-Different

[1]A temporary price based on the manufacturer's requested ex-factory price is determined by the Pricing Committee (Preiskommission, PK) and a price re-evaluation is carried out every 6 months. If the criteria are not met at the second re-evaluation (after 12 months), the EU average price is established on the basis of the information available, i.e. the available countries. In case this price is found to be lower than the temporary price, any excess costs incurred due to the price difference must be paid back to the health insurance.

Country	Population[i]	GDP per capita, current prices, current purchasing power parities (Euro) 2012[ii]	ERP application	Use of ERP	National legal framework related to ERP	Products regulated by ERP	Number of countries in basket	Price level taken into account for reference purposes	Reference price calculation	Rules adopted in case the price is not approved in all reference countries	Price revisions	Rules to choose pack sizes/dosages/pharmaceutical formulations as reference
												pharmaceutical formulations used as reference only when the same reference is not approved in at least 2 countries
Belgium[2]	11,094,850 (2012)	34,000	Yes	Supportive criterion	Yes<br>Law: Law regarding compulsory insurance healthcare and indemnities, coordinated on 14 July 1994 (revised)<br>Program Law of 27 December 2012 which contains most of the measures executing the budget 2013 plan and introducing price cuts based on international prices for reimbursed patented medicines<br><br>Decree: 2 Ministerial Decrees, both of 29 December 1989, one for reimbursement and one for non-reimbursable pharmaceuticals	Brand-only products	26 EU MS	Ex-factory price	Most commonly applied methodologies: Average prices of reference countries or price in the country of origin	Price based on reference countries where price is approved	No	-When different pack sizes/dosages are approved in the reference countries at different prices, the averages price per unit/per dosage unit are used as reference, respectively<br>-Only similar pharmaceutical formulations are used as reference (e.g. oral solid forms are not compared to injectable forms)
Bulgaria	7,282,041 (2013)	5,400	Yes	Main criterion	Yes<br>Law: Medicinal Products in Human Medicine Act, (Promulgated, State Gazette No. 31/13.04.2007, amended)- Chapter	Prescription-only medicines	12 EU MS	Ex-factory price	Lowest price of reference countries	Price based on alternative reference countries: Belgium, Czech Republic,	Yes (Every 6 months)	Not specified

[2] ERP is used as supportive to the pricing decision but the 2013 healthcare budget introduced price cuts based on international prices for reimbursed patented medicines that were on the market for at least 5 years. The prices of these drugs are compared to the prices in six European countries (Austria, Finland, France, Germany, Ireland and the Netherlands). A price average is calculated and a price reduction is proposed for these drugs in Belgium, except if this leads to a lower price than the lowest unit price in any of the six reference countries. For the 2013 exercise, the pharmaceutical company could either accept this price reduction or propose another price reduction, having the same budget impact.

Country	Population[i]	GDP per capita, current prices, current purchasing power parities (Euro) 2012[ii]	ERP application	Use of ERP	National legal framework related to ERP	Products regulated by ERP	Number of countries in basket	Price level taken into account for reference purposes	Reference price calculation	Rules adopted in case the price is not approved in all reference countries	Price revisions	Rules to choose pack sizes/dosages/pharmaceutical formulations as reference
					Twelve "Regulation of the prices of medicinal products" (Title amended, State Gazette No. 102/2012, effective 21.12.2012) Decree: Council of Ministers Decree N 295/2007 (OJ 104, 2007) for adopting a regulation on the terms and conditions for regulation and registration of the prices of medicines [3]					Poland, Latvia and Hungary		
Croatia	4,412,137 (2011)	10,300	Yes	Main criterion	Yes<br>Decree: Ordinance on the criteria for determining the price of a wholesale and how reporting on wholesale prices (OG 155/09)	Reimbursed medicines (in-patent /off-patent)	3 EU MS	Pharmacy purchasing price	-For original products: 90% of the average price of reference countries.<br>-For original breakthrough products: up to 100% of average price of reference countries<br>-For generic products	Price determined with minimum 2 reference countries Alternative reference countries: Spain and Czech Republic	Yes (Annually in February)	-When different pack sizes/dosages are approved in the reference countries at different prices, the same pack size/dosage of the assessed drug are used as reference<br>-If the same pack size is not available, the closest pack size of the assessed drug is used as reference<br>-If the same pharmaceutical formulation is not available in one reference country, a similar pharmaceutical form could be used as reference

[3] According to amendment in price regulation of medicinal products dated of December 2012, the Council of Ministers, on a proposal submitted by the Minister of Health, shall stipulate with an Ordinance the conditions and the rules for regulating the prices of the medicinal products subject to medical prescription, as well as the conditions and the rules for registering the prices of the medicinal products dispensed without medical prescription. This ordinance is not yet published on the website of the Bulgarian Drug Agency.

Country	Population[i]	GDP per capita, current prices, current purchasing power parities (Euro) 2012[ii]	ERP application	Use of ERP	National legal framework related to ERP	Products regulated by ERP	Number of countries in basket	Price level taken into account for reference purposes	Reference price calculation	Rules adopted in case the price is not approved in all reference countries	Price revisions	Rules to choose pack sizes/dosages/pharmaceutical formulations as reference
									up to 70% of average price of reference countries and/or up to 90% of the price of the last bioequivalent generic introduced to the list			
Cyprus	862,011 (2012)	21,100 (2011)	Yes	Main criterion	Yes<br>Decree: All rules for pricing are included in the Ministerial Council Decision of 9/9/2004, (Decision Number 61298). The last revised version has been published in the Government Gazette on 1/10/2012 (Regulatory Administrative Acts 410/2012)	Prescription-only brand medicines	4 EU MS<br>Basket of countries includes one country with high prices (Sweden), two countries with medium prices (Austria and Franc	Pharmacy purchasing price	Average price of reference countries	When a product is not available in the reference countries, there are alternative countries: Denmark and Germany (high), Italy and Belgium (medium), Spain and Portugal (low)<br>When there are no data in some of the selected countries, the price is set based on the rest	Yes (For new products revision of the basket of the available prices is performed annually for the first two years /For old products this revisi	-When different pack sizes are approved in the reference countries at different prices, the closest pack size of the assessed drug is used as reference<br>-When different dosages are approved in the reference countries at different prices, the same dosage of the assessed drug is used as reference<br>-When the pharmaceutical formulation of assessed drug approved in the reference countries is different from the formulation of the assessed drug, no other pharmaceutical formulation is used as reference

Country	Population[i]	GDP per capita, current prices, current purchasing power parities (Euro) 2012[ii]	ERP application	Use of ERP	National legal framework related to ERP	Products regulated by ERP	Number of countries in basket	Price level taken into account for reference purposes	Reference price calculation	Rules adopted in case the price is not approved in all reference countries	Price revisions	Rules to choose pack sizes/dosages/pharmaceutical formulations as reference
							e) and one with low prices (Greece)			of the available data. Price is determined with data from a maximum of four countries and with the minimum of one country When no data is available for products, data from the country of origin is used	on is performed every 2 years)	
Czech Republic	10,516,125 (2013)	14,500	Yes	Main criterion	Yes<br>Law: Public health insurance law no.48/1997 Coll., amended	Reimbursed medicines[4]	For maximum price: 19 EU MS<br>For reimbursement price: all EU MS	Ex-factory price	-For calculating the maximum price: average price of the three lowest prices of reference countries<br>-For calculati	For calculating the maximum price: If the pharmaceutical (with the exception of highly innovative drugs) is not on the	Yes (At least once every three years)	For pricing: Price of a product with the same pack size/strength is used as reference. The price is set as an average of the 3 lowest ex-factory prices, if applicable. If not, price agreement between the manufacturer and the insurance company could be accepted.<br>If none of the first two options can be

[4] A specific group of pharmaceuticals (where there are at least 4 MAHs on the market, at least 4 products with the same route of administration and during where there was no significant increase in prices in the previous 12 months) are regulated only by digressive mark-up scheme and not by fixed maximum price.

Country	Population[i]	GDP per capita, current prices, current purchasing power parities (Euro) 2012[ii]	ERP application	Use of ERP	National legal framework related to ERP	Products regulated by ERP	Number of countries in basket	Price level taken into account for reference purposes	Reference price calculation	Rules adopted in case the price is not approved in all reference countries	Price revisions	Rules to choose pack sizes/dosages/pharmaceutical formulations as reference
									ng basic reimbursement price: lowest price of all drugs from therapeutic group, from the entire EU	market in at least three reference basket states, agreed price (i.e. price agreed between the MAH and the payer) of the pharmaceutical can be used in the evaluation.<br>If not applicable, the price is set as the maximum ex-factory price of the closest therapeutically comparable pharmaceutical available		followed, price is set as the lowest ex-factory price of therapeutically comparable product[5] authorized in the Czech Republic or in reference basket countries<br>For reimbursement: The reimbursement price (in reference group) is based on the lowest ex-factory price of any product in the reference group in all EU countries

[5] Therapeutically comparable product is considered as product with the same active substance, form, strength and package size; If there is no such a product it is accepted products with deviation range of the pack size (deviation of ±10% or higher), with the same active ingredients but different strength etc.

Country	Population[i]	GDP per capita, current prices, current purchasing power parities (Euro) 2012[ii]	ERP application	Use of ERP	National legal framework related to ERP	Products regulated by ERP	Number of countries in basket	Price level taken into account for reference purposes	Reference price calculation	Rules adopted in case the price is not approved in all reference countries	Price revisions	Rules to choose pack sizes/dosages/pharmaceutical formulations as reference
										in the Czech Republic or in the reference basket countries<br>For highly innovative drugs the ex-factory price can be set as the average manufacturer's price found in at least 2 reference basket states		
Denmark	5,602,628 (2013)	43,800	Yes	Main criterion	Yes<br>Agreement between the Danish government and the Danish Association of the Pharmaceutical Industry (LIF) - December 2012	Hospital-only medicines	9 EU MS	Pharmacy purchasing price	Average price of reference countries	Price based on reference countries where price is approved<br>The average price is estimated regardless of how many countries in which the product is marketed.<br>Price ceiling is	Not specified	Not specified

Country	Population[i]	GDP per capita, current prices, current purchasing power parities (Euro) 2012[ii]	ERP application	Use of ERP	National legal framework related to ERP	Products regulated by ERP	Number of countries in basket	Price level taken into account for reference purposes	Reference price calculation	Rules adopted in case the price is not approved in all reference countries	Price revisions	Rules to choose pack sizes/dosages/pharmaceutical formulations as reference
										established only when the product is marketed in at least three of the reference countries		
Estonia	1,286,479 (2013)	13,000	Yes	Main criterion	Yes<br>Law: Health Insurance Act - 2002 (amended) Regulations of the Ministry of Social Affairs	Reimbursed innovative medicines	3 EU MS+ country of origin[6]	Ex-factory price	Not defined/ lowest or average of prices of reference countries	Sometimes some other EU countries have been used as alternatives for the referencing, if the data have been available. If the product is rather new and not on the market in any of the reference countries, the situation just has to be accepted as there is no possibility for ERP	Not specified	Not specified
Finland	5,401,267	35,600	Yes	Supportive	Yes<br>Law: Health Insurance	Reimbursed	26 EU	Pharmacy purchasing	No formula	Not applicable	Price decisi	-When different pack

[6] ERP may include all EU Member States, and sometimes some other EU countries have been used as alternatives for the referencing, if the data have been available.

Country	Population[i]	GDP per capita, current prices, current purchasing power parities (Euro) 2012[ii]	ERP application	Use of ERP	National legal framework related to ERP	Products regulated by ERP	Number of countries in basket	Price level taken into account for reference purposes	Reference price calculation	Rules adopted in case the price is not approved in all reference countries	Price revisions	Rules to choose pack sizes/dosages/pharmaceutical formulations as reference
	(2012)			criterion	Act (1224/2004) (amended)	medicines (except the medicines entering the reference price system directly)	MS+ Iceland, Norway, Liechtenstein	price	(prices in reference countries are one criterion among many others that are considered when approving the reasonable wholesale price)		on in force for a maximum of five years (3 years for new active pharmaceutical ingredient)<br>ERP used as a part of the renewal application	sizes/dosages/pharmaceutical formulations are approved in the reference countries at different prices, the same pack size/dosage/formulation of the assessed drug are used as reference<br>-If the prices of the same pack size/dosage/formulation are not available, the closest pack size/dosage/formulation of the assessed drug could be used as reference
France	64,350,226 (2009)	31,100	Yes	Main criterion	Yes<br>Framework agreement signed between LEEM and CEPS - 05 December 2012<br>Law: Art. L-162-17-6 of the Social Security Code	Innovative reimbursed medicines[7]	4 EU MS	Ex-factory price	Prices similar to those in the reference countries	Not specified	Yes (Innovative drugs under ERP system benefit from a	Not specified

[7] Innovative patented drugs in ambulatory settings and innovative hospital drugs that are reimbursed directly by statutory health insurance
• Drug having a level of improvement of clinical benefit (ASMR) I, II or III for their principal indication.
• Drug having a level of improvement of clinical benefit (ASMR) IV in specific cases:
- The daily cost of the treatment does not exceed that of the comparator. However a price notification of a drug having higher daily cost than the comparator could be accepted if the manufacturer demonstrates that the drug will generate cost savings of a same amount for the health insurance system.
-The drug will not replace a generic drug, or one which will shortly face generic competition.
ERP may also be used as an argument during negotiations with the industry for dugs with ASMR IV or V.

Country	Population[i]	GDP per capita, current prices, current purchasing power parities (Euro) 2012[ii]	ERP application	Use of ERP	National legal framework related to ERP	Products regulated by ERP	Number of countries in basket	Price level taken into account for reference purposes	Reference price calculation	Rules adopted in case the price is not approved in all reference countries	Price revisions	Rules to choose pack sizes/dosages/pharmaceutical formulations as reference
											European price guarantee of 5 years with a one-year extension in specific cases)	
Germany	81,843,743 (2012)	32,600	Yes	Supportive criterion	Yes<br>Law: AMNOG (Arzneimittelmarktneuordnungsgesetz) Healthcare Reform - in effect since 01 January 2011	Innovative reimbursed medicines[8]	15 EU MS	Ex-factory price	Not clearly defined<br>The price range is set by European reference prices as upper and appropriate comparators price as lower level.<br>Drug sales	Not specified	Not specified	Not specified

[8]Pharmaceuticals that demonstrate a clinical added value will be subject to price negotiations between the Federal Association of Sickness Funds and the pharmaceutical company, in consultation with the Association of Private Health Insurance Companies. ERP is one of the criteria for setting the reimbursement price. In case companies fail to negotiate a price discount with health insurers for their new drugs, an arbitration body – consisting of representatives of the sickness funds, the pharmaceutical industry and neutral members – has three months to set a price that takes into consideration international prices.

Country	Population[i]	GDP per capita, current prices, current purchasing power parities (Euro) 2012[ii]	ERP application	Use of ERP	National legal framework related to ERP	Products regulated by ERP	Number of countries in basket	Price level taken into account for reference purposes	Reference price calculation	Rules adopted in case the price is not approved in all reference countries	Price revisions	Rules to choose pack sizes/dosages/pharmaceutical formulations as reference
									and purchasing power parity in each of the 15 reference countries must be taken into account			
Greece	11,309,885 (2011)	19,900 (2007)	Yes	Main criterion	Yes<br>Laws: 3790/2009 (pricing method) - September 2009. Ministerial Decision no. ΔΥΓ3 (α) /οικ. 7789 : "Provisions re pricing of medicines"	Brand-only products	22 EU MS	Ex-factory price	Average of the 3 lowest prices of reference countries	Medicinal product should have been priced in at least 3 reference countries	Yes (Prices may be updated up to 4 times a year)	-When different pack sizes/dosages are approved in the reference countries at different prices, the same pack size/dosage of the assessed drug are used as reference<br>-If the same pack size/dosage is not available in a reference country, the price is extrapolated based on the price of the other pack sizes/dosages according to a correlation provided in Article 12 of Ministerial Decision no ΔΥΓ3(α) /οικ. 7789. When two or more strengths of the same drug are priced and the prices derived are disproportional to each other, the lowest price shall be taken<br>-Nothing specified

Country	Population[i]	GDP per capita, current prices, current purchasing power parities (Euro) 2012[ii]	ERP application	Use of ERP	National legal framework related to ERP	Products regulated by ERP	Number of countries in basket	Price level taken into account for reference purposes	Reference price calculation	Rules adopted in case the price is not approved in all reference countries	Price revisions	Rules to choose pack sizes/dosages/pharmaceutical formulations as reference
												regarding different pharmaceutical formulations
Hungary	9,985,722<br>(2011)	9,800	Yes	Main criterion	Yes<br>Law: Act XCVIII - 2006 on General Provisions relating to the Reliable and Economically Feasible Supply of Medicinal Products and Medical Appliances and on the Distribution of Medicinal Products	New active substances	27 EU MS+ Iceland, Norway, Lichtenstein<br>In practice Switzerland referenced as well	Ex-factory price	Lowest price of reference countries	Price based on reference countries where price is approved if at least 3 reference countries reimburse the product	Yes (At least once a year, however, price comparisons are not reviewed on a regular basis)	-When different pack sizes are approved in the reference countries at different prices, the closest pack size of the assessed drug is used as reference<br>-When different dosages are approved in the reference countries at different prices, the same dosage of the assessed drug is used as reference<br>-When the pharmaceutical formulation of assessed drug approved in the reference countries is different from the formulation of the assessed drug, the closest pharmaceutical form is used as reference but only formulation that is administered in the same way (e.g. tablets vs. film-coated tablets is accepted; tablets vs. oral suspension/injection is not accepted)

Country	Population[i]	GDP per capita, current prices, current purchasing power parities (Euro) 2012[ii]	ERP application	Use of ERP	National legal framework related to ERP	Products regulated by ERP	Number of countries in basket	Price level taken into account for reference purposes	Reference price calculation	Rules adopted in case the price is not approved in all reference countries	Price revisions	Rules to choose pack sizes/dosages/pharmaceutical formulations as reference
Iceland	319,575 (2012)	32,900	Yes	Main criterion	Yes<br>Decrees: Regulation related to the Icelandic Medicine Pricing and Reimbursement Committee n°353/2013 - 27 March 2013<br>Medicinal Products Act no. 93/1994 - Article 43	Prescription-only medicines (in-patent/off-patent, including parallel imported products and hospital products)	3 EU MS+ Norway	Pharmacy purchasing price	-Original products: Price is compared to the average price on the corresponding original product of reference countries<br>-Generic products: Price is compared to the average price of the corresponding generic of reference countries<br>-Parallel imported products: Price should be lower than price on the corresponding original or generic product	Price based on reference countries where price is approved (one country or more)	Yes (At least every 2 years- It starts in January till complete revision (April-May-June-July))	-When different pack sizes are approved in the reference countries at different prices, the same package size, plus-minus 10% of the assessed drug is used as reference<br>-When different dosages are approved in the reference countries at different prices, the same dosage of the assessed drug is used as reference<br>-When the pharmaceutical formulation of assessed drug approved in the reference countries is different from the formulation of the assessed drug, only comparable pharmaceutical formulations are used as reference (e.g. tablet versus capsules)

Country	Population[i]	GDP per capita, current prices, current purchasing power parities (Euro) 2012[ii]	ERP application	Use of ERP	National legal framework related to ERP	Products regulated by ERP	Number of countries in basket	Price level taken into account for reference purposes	Reference price calculation	Rules adopted in case the price is not approved in all reference countries	Price revisions	Rules to choose pack sizes/dosages/pharmaceutical formulations as reference
									in Iceland -Hospital product: Price may not exceed the lowest price of reference countries, this includes innovative medicines			
**Ireland**	4,467,854 (2010)	35,700	Yes	Main criterion	Yes The 2012 Framework agreement between the Irish Pharmaceutical Healthcare Association Ltd and the Department of Health and the Health Service Executive on the Supply Terms, Conditions, and Prices of Medicines - in effect since November 1st 2012.	Reimbursed prescription-only medicines	9EU MS	Ex-factory price	Currency adjusted average price of reference countries	Price based on reference countries where price is approved and revised when a new price is available in an additional country<br>If a new medicine is not available in any of the nominated EU states, the Irish price to wholesaler is agreed between	Yes (Planned by the framework agreement - signed for a duration of 3 years- at defined dates (November 2012 and January	Not specified

Country	Population[i]	GDP per capita, current prices, current purchasing power parities (Euro) 2012[ii]	ERP application	Use of ERP	National legal framework related to ERP	Products regulated by ERP	Number of countries in basket	Price level taken into account for reference purposes	Reference price calculation	Rules adopted in case the price is not approved in all reference countries	Price revisions	Rules to choose pack sizes/dosages/pharmaceutical formulations as reference
										representatives of the manufacturer/ importer concerned and the Health Service Executive within 90 days of the date of the reimbursement application	2013 depending on products): downward price realignment based on the currency-adjusted average ex-factory price of the drug in the reference countries in which the medicine is available)	
Italy	60,626,442 (2011)	25,700	Yes	Supportive criterion	Yes<br>Law: no. 326 - November 24, 2003<br>Decree: Interministerial Committee for Economic Planning (Comitato Interministeriale per la Programmazione Economia – CIPE) Resolution – February	Reimbursed medicines (in-patent/off-patent)	27 EU MS	Ex-factory/Pharmacy purchasing price/Pharmacy retail price	Only used as additional information during the price negotiation procedu	Price based on reference countries where price is approved	Yes (Prices are set for an initial 2-year period)	-When different pack sizes are approved in the reference countries at different prices, the closest package size of the assessed drug is used as reference<br><br>-When different

Country	Population[i]	GDP per capita, current prices, current purchasing power parities (Euro) 2012[ii]	ERP application	Use of ERP	National legal framework related to ERP	Products regulated by ERP	Number of countries in basket	Price level taken into account for reference purposes	Reference price calculation	Rules adopted in case the price is not approved in all reference countries	Price revisions	Rules to choose pack sizes/dosages/pharmaceutical formulations as reference
					1st 2001<br>Law 122/2010 (DL 78/2010) for generic drugs placed in class A				re considering lowest price but also GDP and pharmaceutical market size of reference countries<br>For generic drugs placed in class A, AIFA set a maximum reimbursement price for package, equal to active ingredient, dosage form and route of administration, based on recognition of the current prices in four EU countries that are closer to			dosages are approved in the reference countries at different prices, the same dosage or the closest dosage of the assessed drug is used as reference<br>-When the pharmaceutical formulation of assessed drug approved in the reference countries is different from the formulation of the assessed drug, no other pharmaceutical formulation is used as reference

Country	Population[i]	GDP per capita, current prices, current purchasing power parities (Euro) 2012[ii]	ERP application	Use of ERP	National legal framework related to ERP	Products regulated by ERP	Number of countries in basket	Price level taken into account for reference purposes	Reference price calculation	Rules adopted in case the price is not approved in all reference countries	Price revisions	Rules to choose pack sizes/dosages/pharmaceutical formulations as reference
									the Italian context: United Kingdom, Germany, Spain and France [9]			
Latvia	2,248,374 (2010)	10,900	Yes	Main criterion	Yes<br>Decree: Regulation of the Cabinet of Ministers of the Republic of Latvia no. 899 - 31 October 2006	Reimbursed medicines	7 EU MS<br>Regulation also refers to a price comparison in other EU MS	Ex-factory price/Pharmacy purchasing price	Price for the medicinal products or medical devices to be included on the List of reimbursable medicinal products: shall not be higher than the third lowest manufacturer's sales prices or wholesale prices for these medicinal products in Czech	Price based on reference countries where price is approved and revised when new price available in additional country	Yes (Prices for the medicines included in the Positive list are verified once in 2 years)	-When different pack sizes are approved in the reference countries at different prices, the same or closest package size of the assessed drug is used as reference<br>-When different dosages are approved in the reference countries at different prices, it is considered as a situation where the drug is not available<br>-When the pharmaceutical formulation of assessed drug approved in the reference countries is different from the formulation of the assessed drug, no other pharmaceutical formulation is used as reference

[9] From April 2011, reimbursed off-patent originals and generics prices were realigned to average prices in France, Germany, Spain and the United Kingdom, in addition to the lowest-priced product in the cluster. Where the reference prices exceed the mean European reference price, they are cut to the European level.

Country	Population[i]	GDP per capita, current prices, current purchasing power parities (Euro) 2012[ii]	ERP application	Use of ERP	National legal framework related to ERP	Products regulated by ERP	Number of countries in basket	Price level taken into account for reference purposes	Reference price calculation	Rules adopted in case the price is not approved in all reference countries	Price revisions	Rules to choose pack sizes/dosages/pharmaceutical formulations as reference
									Republic, Denmark, Romania, Slovakia and Hungary, and shall not exceed the manufacturer's sales prices or wholesale prices for these medicinal products in Estonia and Lithuania			
Lithuania	2,971,905 (2013)	11,000	Yes	Main criterion	Yes<br>Decrees:<br>Governmental Decree No 1806 of 23 December, 2009 on calculation of base (reimbursed) prices of medicinal product. The reference countries are set by Governmental Decree No 256 of 10 March, 2010	Reimbursed medicines	8 EU MS	Price declared by marketing authorisation holder or his/her representative for Lithuania<br>Legal acts do not stipulate special requirements what type of price (ex-factory, PPP or PRP) shall be declared by manufacture	Reference price shall not exceed 95% of the average price of reference countries	Price based on reference countries where price is approved.<br>If no data on prices in the reference countries, price based on price in the country of origin	Yes (Annually/In the first quarter of the year before adoption of the Annual Price List)	-When different pack sizes/dosages are approved in the reference countries at different prices, the same pack size/dosage of the assessed drug are used as reference<br>-If the same pack size/dosage are not available, the closest pack size/dosage of the assessed drug is used as reference (and comparatively cheapest package)<br>-When the pharmaceutical

Country	Population[i]	GDP per capita, current prices, current purchasing power parities (Euro) 2012[ii]	ERP application	Use of ERP	National legal framework related to ERP	Products regulated by ERP	Number of countries in basket	Price level taken into account for reference purposes	Reference price calculation	Rules adopted in case the price is not approved in all reference countries	Price revisions	Rules to choose pack sizes/dosages/pharmaceutical formulations as reference
								r for reference purpose.				formulation of assessed drug approved in the reference countries is different from the formulation of the assessed drug, no other pharmaceutical formulation is used as reference
Luxembourg	537,039 (2013)	83,600	Yes	Main criterion	Yes<br>Decree: Grand-Ducal Regulation of 1 December 2011 laying down the criteria, conditions and procedures relating to the pricing of medicinal products for human use	All medicines	Country of origin	Pharmacy retail price	Price cannot be greater than the price granted by the competent authority of the country of origin	Not applicable: origin of the drug taken into consideration	Yes (Pharmaceutical company required to report any change in price of a presentation in the country of origin, within one month of the change)	Not specified
Malta	421,230 (2013)	16,300	Yes	Main criterion		-Public Sector: Reimbursed medicines<br>-	Public sector: 11 EU MS<br>Priva	-Public sector: Pharmacy purchasing price<br>-Private	-Public Sector: average price of reference countries	Price based on reference countries where price is approved	Yes (Public Sector: variable perio	Public sector:<br>-When different pack sizes are approved in the reference countries at different prices, the cheapest pack size per unit of the assessed drug is

Country	Population[i]	GDP per capita, current prices, current purchasing power parities (Euro) 2012[ii]	ERP application	Use of ERP	National legal framework related to ERP	Products regulated by ERP	Number of countries in basket	Price level taken into account for reference purposes	Reference price calculation	Rules adopted in case the price is not approved in all reference countries	Price revisions	Rules to choose pack sizes/dosages/pharmaceutical formulations as reference
					-Public sector : Yes Law: The Availability of Medicinal Products within the Government Health Services Regulations (L.N. 58 - 2009) within the Medicines Act (Cap. 458) Moreover, there is an internal Standard Operating Procedure related to use of ERP<br><br>-Private Sector: No.ERP operates under the provisions of a voluntary agreement	Private Sector: Medicines sold in community pharmacies	te sector: 12 EU MS	sector: Pharmacy retail price	-Private Sector: Reference countries are categorized in a three-tier price-level classification using the Harmonized Index of Consumer Prices (HICP) for the health sector Low-priced tier (L): Spain, United Kingdom, Portugal, France Medium-priced tier (M): Belgium, Iceland, Cyprus, Italy High-priced tier (H): Denmark , Germany, Ireland,		d/Private Sector: every 1½ - 2 years or as required if triggered by a consumer complaint)	used as reference -When different dosages are approved in the reference countries at different prices, the same dosage of the assessed drug is used as reference -When the pharmaceutical formulation of assessed drug approved in the reference countries is different from the formulation of the assessed drug, no other pharmaceutical formulation is used as reference <u>Private sector</u>: The EPR entails a strict like-with-like comparison of the basis of the proprietary name, strength and pharmaceutical formulation combination. However weighted reference prices of pack sizes in the range of ½ to twice the local pack size are used

Country	Population[i]	GDP per capita, current prices, current purchasing power parities (Euro) 2012[ii]	ERP application	Use of ERP	National legal framework related to ERP	Products regulated by ERP	Number of countries in basket	Price level taken into account for reference purposes	Reference price calculation	Rules adopted in case the price is not approved in all reference countries	Price revisions	Rules to choose pack sizes/dosages/pharmaceutical formulations as reference
									Norway An algorithm is used for the derivation of a reference price			
Norway	5,051,275 (2013)	77,500	Yes	Main criterion	Yes<br>Law: LOV 1992-12-04 nr 132: Lov om legemidler (Norwegian Act on Medicinal Products). In addition to these rules, the Norwegian Medicines Agency (NOMA) also uses the Guideline on Pricing of Medicinal Products when determining the price	Prescription-only medicines (in-patent)	9 EU MS	Pharmacy purchasing price	Average of the 3 lowest prices of reference countries [10]	Price based on reference countries where price is approved	Yes (Annually- In the end of August/early September each year, NOMA publishes the active ingredients that will be reconsidered, and in what order	-When different pack sizes are approved in the reference countries at different prices, the same pack size of the assessed drug is used as reference<br>-When pack size are not comparable, price comparisons with other countries are done on the basis of units (price per tablet, dose, etc.)[12]<br>-When different dosages are approved in the reference countries at different prices, price ratios between different strengths are considered[13]<br>-When the pharmaceutical formulation of assessed drug approved in the reference countries is different from the

[10] In some situations, when using the general rules, the calculated price may drop to a price level which is so low that it may be expedient to set a higher price. Two conditions must apply to justify deviation from the main rules:
1. There is a major risk that the medicine will no longer be available in the market if the calculated maximum price is implemented.
2. The absence of the medicine from the market could have negative consequences for the availability of cost-effective medicines.
If these conditions apply, Norwegian Medicines Agency (NOMA) will consider setting a higher price based on discretionary judgment.

Country	Population[i]	GDP per capita, current prices, current purchasing power parities (Euro) 2012[ii]	ERP application	Use of ERP	National legal framework related to ERP	Products regulated by ERP	Number of countries in basket	Price level taken into account for reference purposes	Reference price calculation	Rules adopted in case the price is not approved in all reference countries	Price revisions	Rules to choose pack sizes/dosages/pharmaceutical formulations as reference
											for the upcoming year)[11]	formulation of the assessed drug, different varieties of the same product will be considered as varieties of the same pharmaceutical (e.g. tablets, capsules, melting tablets, soluble tablets and effervescent tablets). NOMA will only set a higher price for other varieties of the same medicine on exception
**Poland**	38,533,299 (2013)	9,600 (2011)	Yes	Supportive criterion	Yes<br>Law: Act dated 12 May 2011 related to the reimbursement of medicines, foodstuffs for particular nutritional use and medical devices.<br>But no legal rules nor informal guidelines on ERP use	Reimbursed medicines	27 EU MS+ Norway, Iceland, Liechtenstein, Switzerland	Ex-factory price	The Economic Committee takes into account during the price negotiations with the manufacturer, the	Not applicable (Price not based on ERP)	No (Prices are not ERP based thus ERP is not a basis for further revisions-	The company submits data only on the same drug for reimbursement of which it applies

[12] When setting the price, differentiation is normally made between the price per unit in large (generally defined as more than 30 units) and small packages (generally defined as 30 or fewer units), with some exemptions for some medicinal products. In some cases, when comparing prices from different countries, the price per tablet in a small package may be lower than the price per tablet in a large package. In such cases, the price per tablet in the large package is set equal to the price per tablet in the small package. If the price per tablet is higher in a small package than in a large package, the price difference is accepted provided that the difference is not considered unreasonable.

[13] When setting the price, the Norwegian Medicines Agency (NOMA) will aim at a reasonable price ratio between different strengths of a given product. A low dosage may not have a higher maximum price than a higher dosage.

[11] The Norwegian Medicines Agency (NOMA) yearly re-evaluates the maximum price for the 250 active ingredients with the highest turnover. This is done to ensure that the maximum prices reflect the developments in European prices. The prices of newly-launched products are exempt from this rule. In the two year period after a launch, NOMA may request information about new prices every six months from the Market Authorisation Holder (MAH) in question. Withdrawal of a product from one of the reference countries may be cause for an alteration in the price in Norway. Documentation must be produced to show that a product has in fact been withdrawn from the market if this is to give cause for price changes.

Country	Population[i]	GDP per capita, current prices, current purchasing power parities (Euro) 2012[ii]	ERP application	Use of ERP	National legal framework related to ERP	Products regulated by ERP	Number of countries in basket	Price level taken into account for reference purposes	Reference price calculation	Rules adopted in case the price is not approved in all reference countries	Price revisions	Rules to choose pack sizes/dosages/pharmaceutical formulations as reference
									maximum and minimum ex-factory prices over the preceding year in the EU and EFTA MS where the drug is already reimbursed		reimbursement status is valid for a 2-5 years period)	
**Portugal**	10,637,713 (2010)	16,300 (2010)	Yes	Main criterion	Yes<br>Laws: Decree- law 112/2011 - 9 November 2011, amended by Decree-law 152/2012 - 12 July 2012<br>Ordinance 4/2012 - 02 January 2012<br>Decree-law 34 - 27 February 2013<br>Ordinance 91/2013	Prescription-only medicines and reimbursed OTC (excluding generics and the hospital restricted prescription medici	3 EU MS	Ex-factory price	Average price of reference countries	Price based on reference countries where price is approved[14]	Yes (Annually based on the average price of the same drug in the reference countries/ Fixed period defin	-When different pack sizes/dosages are approved in the reference countries at different prices, the same pack size/dosage of the assessed drug are used as reference<br>-If the same pack size/dosage are not available, the closest pack size/dosage of the assessed drug are used as reference<br>-When the pharmaceutical formulation of assessed drug approved in the

[14] -In case that the same pharmaceutical does not exist in any reference countries, it is used the average of the lowest ex-factory prices of the identical or similar pharmaceuticals in the reference countries (excluding generics).
-In case that neither identical nor similar pharmaceuticals exist in any reference country but exist in Portugal, it is used the ex-factory price of the identical or similar pharmaceuticals that are commercialised in the national market.
-In case that neither identical nor similar pharmaceuticals exist in any reference country nor in Portugal, the ex-factory price of the original country is used.
The price of the drug is considered as provisional, if it was not based on the price of the same medicine or, if not available, identical or similar pharmaceuticals in the reference countries. This price will be provisional up to the drug price may be based on the price of the same medicine or, if not available, identical or similar pharmaceuticals in 2 of the 3 reference countries.

Country	Population[i]	GDP per capita, current prices, current purchasing power parities (Euro) 2012[ii]	ERP application	Use of ERP	National legal framework related to ERP	Products regulated by ERP	Number of countries in basket	Price level taken into account for reference purposes	Reference price calculation	Rules adopted in case the price is not approved in all reference countries	Price revisions	Rules to choose pack sizes/dosages/pharmaceutical formulations as reference
						nes)					ed each year)	reference countries is different from the formulation of the assessed drug, no other pharmaceutical formulation is used as reference
**Romania**	21,462,186 (2010)	6,200	Yes	Main criterion	Yes<br>Order no. 75 - 30 January 2009 approving the Norms regarding the calculation of the prices of medicinal products for human use	Prescription-only medicines	12 EU MS	Ex-factory price	Lowest price of reference countries	If there is no price in the 12 EU MS then the price from the origin country is considered	Yes (Annually in April)	-When different pack sizes are approved in the reference countries at different prices, the same pack size of the assessed drug is used as reference<br>-If the same pack size/dosage are not available, the closest pack size of the assessed drug is used as reference<br>-No specific information for different dosages and pharmaceutical formulations
**Slovakia**	5,410,836 (2013)	13,200	Yes	Main criterion	Yes<br>Law: Act no. 363/2011 Coll., on the conditions of reimbursing drugs, medical devices and dietetic foods from public health insurance revised January 2013.	Reimbursed medicines	27 EU MS	Ex-factory price	Average price of the 3 lowest prices from all EU MS	Price based on reference countries where price is approved	Yes (Twice a year in April and October)	-When different pack sizes/dosages are approved in the reference countries at different prices, the same pack size/dosage of the assessed drug are used as reference<br>-When the pharmaceutical formulation of assessed drug approved in the reference countries is different from the formulation of the assessed drug, no other pharmaceutical

Country	Population[i]	GDP per capita, current prices, current purchasing power parities (Euro) 2012[ii]	ERP application	Use of ERP	National legal framework related to ERP	Products regulated by ERP	Number of countries in basket	Price level taken into account for reference purposes	Reference price calculation	Rules adopted in case the price is not approved in all reference countries	Price revisions	Rules to choose pack sizes/dosages/pharmaceutical formulations as reference
												formulation is used as reference
Slovenia	2,058,821 (2013)	17,200	Yes	Main criterion for Maximum Allowed Price/ Supportive criterion for Extraordinary Higher Price[15]	Yes<br>Law: Medicines Act- Official Gazette of RS, no. 31/2006 and 45/2008<br>Decree: Rules on the pricing of medicinal products for human use-Official Gazette of RS, no. 102/2010<br>Pricing Regulation: Official Gazette of RS, no. 6/12	Reimbursed medicines (in-patent /off-patent)	3 EU MS	Ex-factory price	-For original drugs and biosimilars: the maximum price is based on the lowest price of the same drug in reference countries and shall not exceed 100% of the calculated price<br>-For generics: the maximum price is based on the average of the mean values calculated in each country	Price based on reference countries where price is approved<br>-For original drugs and generics:<br>*If the drug is not available in the reference countries, the comparison of prices is based on the medicines prices in other EU MS and EEA countries where the drug is marketed<br>*If the drug is not available in EU MS and EEA countries, the compariso	Yes (Twice a year in case of changes in the price of countries of reference in March including 1 April and September including 1 October)	-When different pack sizes/dosages are approved in the reference countries at different prices, the same pack size/dosage of the assessed drug are used as reference<br>-In case the same pack size is not available, a price per unit is calculated[16]<br>-In case the same dosage is not available, a price conversion from other dosages is performed. It is applied only in the case when the same dosage is not available in any of the reference MS. In the opposite case, only the products in one or 2 MS that comply directly, are used<br>-If the same pharmaceutical formulation is not available in the reference countries and for a similar route of administration, comparable pharmaceutical

[15] Extraordinary Higher Price is a kind of higher premium price for eligible products.
[16] Pricing Rule contains provisions for proportional calculation of the size of package according to the number of units present, as well as for amount of active principle in particular unit or mass/volume measure of the active principle in a multidosing form (i.e. solutions)

Country	Population[i]	GDP per capita, current prices, current purchasing power parities (Euro) 2012[ii]	ERP application	Use of ERP	National legal framework related to ERP	Products regulated by ERP	Number of countries in basket	Price level taken into account for reference purposes	Reference price calculation	Rules adopted in case the price is not approved in all reference countries	Price revisions	Rules to choose pack sizes/dosages/pharmaceutical formulations as reference
									between the least and most expensive generics and shall not exceed a certain percentage of the calculated price (between 68 and 72% in 2012, depending on the number of reference countries where the same generic drug is marketed)<br>-For drugs application for extraordinary permitted higher prices: ERP is one of the criteria to set the	n of prices is based on prices in other European countries where the drug is marketed<br>-For biosimilars:<br>*If the drug is not available in the reference countries, the comparison of prices is based on the medicines prices in other EU MS and EEA countries where the drug is marketed<br>*If the drug is not available in EU MS and EEA countries, ex-factory price of the original biological medicinal product is taken into account		formulations (e.g. capsule-tablet) can be used as reference. Cross referencing of fast-release and modified-release formulation is not possible

Country	Population[i]	GDP per capita, current prices, current purchasing power parities (Euro) 2012[ii]	ERP application	Use of ERP	National legal framework related to ERP	Products regulated by ERP	Number of countries in basket	Price level taken into account for reference purposes	Reference price calculation	Rules adopted in case the price is not approved in all reference countries	Price revisions	Rules to choose pack sizes/dosages/pharmaceutical formulations as reference
									prices (comparison with ex-factory prices in the EU member States and EEA countries where the product is marketed) among others, and the prices are calculated according to a complex formula as defined per regulation			
Spain	46,152,926 (2011)	22,300	Yes	Supportive criterion	ERP is not more regulated.<br>Nevertheless, ERP respond to internal criteria of the Interministerial pricing committee	All innovative medicines when no comparator available in	Not regulated[17]	Ex-factory price	Lowest price of reference countries	No data available	Yes (Yearly reviews of European prices for selected mark	Not specified

[17] Not regulated, the criteria adopted in the Interministerial Pricing Committee is to consider, in general , countries from the Euro zone due to fluctuating exchange rates in other countries, although the criteria might change.

Country	Population[i]	GDP per capita, current prices, current purchasing power parities (Euro) 2012[ii]	ERP application	Use of ERP	National legal framework related to ERP	Products regulated by ERP	Number of countries in basket	Price level taken into account for reference purposes	Reference price calculation	Rules adopted in case the price is not approved in all reference countries	Price revisions	Rules to choose pack sizes/dosages/pharmaceutical formulations as reference
						Spain					et)	
Sweden	9,555,893 (2013)	43,000	No	Not applicable	Not applicable	Not applicable	Not applicable	Not applicable	Not applicable	Not applicable	Not applicable	Not applicable
Switzerland	7,954,662 (2012)	61,900	Yes	Main criterion	Yes<br>Decree: Ordinance on health insurance services/Ordonnance sur les prestations de l'assurance des soins (OPAS) -Septembre 1995	Reimbursed medicines	6 EU MS	Ex-factory price	Average price of reference countries	Price based on reference countries where price is approved and revised when new price available in additional country	Yes (Every 3 years, review of prices against prices in the reference countries (May))	-When different pack sizes are approved in the reference countries at different prices, the closest pack size of the assessed drug is used as reference<br>-When different dosages are approved in the reference countries at different prices, the same dosage of the assessed drug is used as reference<br>-When the pharmaceutical formulation of assessed drug approved in the reference countries is different from the formulation of the assessed drug, different formulations can exceptionally be used depending on the case
The Netherlands	16,779,575 (2013)	35,800	Yes	Main criterion		Prescription-only medicines (All outpatient	4 EU MS	Pharmacy purchasing price	Average price of reference countries	Price only set if a comparable drug is marketed in at least 2 of the 4	Yes (Twice-yearly basis)	-When different pack sizes are approved in the reference countries at different prices, the cheapest pack size per defined

Country	Population[i]	GDP per capita, current prices, current purchasing power parities (Euro) 2012[ii]	ERP application	Use of ERP	National legal framework related to ERP	Products regulated by ERP	Number of countries in basket	Price level taken into account for reference purposes	Reference price calculation	Rules adopted in case the price is not approved in all reference countries	Price revisions	Rules to choose pack sizes/dosages/pharmaceutical formulations as reference
					Yes<br>Law: the Medicines Prices Act (WGP) - 1996 (amended)	drugs, including branded and generic drugs, and high-cost medicines and orphans drugs for inpatient care)				reference countries		daily dose of the assessed drug is used as reference<br>-When different dosages are approved in the reference countries at different prices, the same dosage of the assessed drug is used as reference<br>-When the pharmaceutical formulation of assessed drug approved in the reference countries is different from the formulation of the assessed drug, no other pharmaceutical formulation is used as reference
UK	62,026,962<br>(2010)	30,500	No	Not applicable	Not applicable	Not applicable	Not applicable	Not applicable	Not applicable	Not applicable	Not applicable	Not applicable

[i] Eurostat. Population
Available from:
http://epp.eurostat.ec.europa.eu/tgm/table.do?tab=table&init=1&language=en&pcode=tps00001&plugin=1
(Cited 2013 Sep 12)

[ii] Eurostat. GDP Available from: http://appsso.eurostat.ec.europa.eu/nui/show.do?dataset=nama_gdp_c&lang=en
(Cited 2013 Sep 13)
